# The importance of layer-dependent molecular twisting for the structural anisotropy of interfacial water

**DOI:** 10.1126/sciadv.adz5505

**Published:** 2026-04-22

**Authors:** Alexander P. Fellows, Louis Lehmann, Álvaro Díaz Duque, Martin Wolf, Roland R. Netz, Martin Thämer

**Affiliations:** ^1^ Fritz-Haber-Institut der Max-Planck-Gesellschaft, Faradayweg 4-6, 14195 Berlin, Germany.; ^2^ Department of Physics, Freie Universität Berlin, Arnimallee 14, 14195 Berlin, Germany.

## Abstract

The unique structural properties of interfacial water are at the heart of many important processes in electrochemistry, climate science, and biophysics. At interfaces, water molecules exhibit preferential orientations and an altered intermolecular H-bond connectivity. Characterizing this layer-dependent anisotropic structure for such a thin molecular boundary, however, is a veritable challenge, with many important details remaining unknown. Here, we combine a novel depth-resolved second-order spectroscopy with molecular dynamics simulations to study the anisotropic structure at the air-water interface through the H─O─H bending vibration. We first uncover the elusive anisotropic interfacial response by removing the bulk-like (quadrupolar) term that is found to dominate the spectrum and has hampered previous experimental investigations of the interfacial structure. Thereafter, we reveal that the molecular structure at the interface shows a pronounced layering of alternating tilt-twist motifs. This highlights the often-disregarded anisotropy in the molecular twist angle and offers a revised picture of aqueous interfaces.

## Teaser

Depth-resolved vibrational spectroscopy and simulations reveal the twisted nature of water molecules at the interface to air.

## INTRODUCTION

Understanding the anisotropic structure of water at aqueous interfaces, and particularly its evolution with depth, is of paramount importance due to its relevance in interfacial processes ranging from physiology, atmospheric chemistry, and electrochemical systems. The sheer presence of the interface induces changes to molecular orientations, diffusion dynamics, and the interconnectivity of the H-bond network (*1*). These structural deviations compared to the isotropic bulk are at the heart of the aforementioned processes; however, revealing their details poses a major challenge.

A common approach for accessing the specific structural details of water is through the assessment of vibrational spectra as the resonant line shapes are highly sensitive to the details of the H-bond connectivity. For example, measurements of bulk water have shown that stronger bonds result in red-shifted O─H stretches and blue-shifted H─O─H bending vibrations (*2*, *3*). In contrast to linear vibrational spectroscopies, which are dominated by these bulk signals, nonlinear optical sum-frequency generation (SFG) spectroscopy can exclusively probe the interfacial region due to its second-order selection rules. These render the responses orientationally dependent under the electric dipole approximation (EDA), leading to cancellation of signals from isotropic regions (*4*–*7*). This unique property can make SFG a selective probe of the different aspects of the out-of-plane anisotropic structure present at the interface, namely, changes in the orientational distribution (preferential orientations) and intermolecular connectivity. Furthermore, provided the phase of the nonlinear response is measured (by phase-resolved SFG), the absolute orientations can also be directly assessed (*8*). Extracting the anisotropic contribution to the interfacial structure is an essential step for understanding the behavior of the interface because it is precisely this structural perturbation from the bulk that defines its non-bulk-like properties. Gaining a clear picture of the anisotropic structure at the interface and especially its depth dependence is, however, not straightforward simply from analyzing SFG spectra. Nevertheless, it can be facilitated by comparing the experimental results with SFG spectra calculated from molecular dynamics (MD) simulations, which can directly yield the different structural motifs present at the interface and can correlate them to the observed resonant features (*9*–*15*).

Many SFG investigations have analyzed the O─H stretching modes of aqueous interfaces, with simulations reproducing the main observed spectral features, including their absolute amplitudes, but still showing slight deviations in the spectral line shape (*13*, *14*, *16*, *17*). From such studies at the pure air-water interface, the presence of “free” O─H species (water molecules with “dangling” bonds) has been identified. Furthermore, both positive and negative spectral contributions at different frequencies are observed, which are typically attributed to “pointing up” and “pointing down” structural motifs with differing H-bond interconnectivity, respectively. Such a description of the molecular directionality is commonly referring to the projection of the molecular dipole onto the surface normal (defining its molecular tilt angle). Overall, this paints a picture of the air-water interface where the topmost molecules are pointing toward air with dangling bonds, followed by molecules in deeper layers preferentially pointing “down” with stronger H-bonding.

Despite these observations, a thorough interpretation of the O─H stretching responses in terms of the molecular structure is difficult. This is due to the presence of two distinct stretching modes on each molecule, either seen as a symmetric and anti-symmetric mode or as two decoupled O─H oscillators, with differing directionality. This, combined with the intrinsically different frequencies of these two modes, muddies the connection between specific spectral features and the molecular and intermolecular structure. Additional complexity then originates from the strong inter- and intramolecular coupling and delocalization of the stretching modes (*18*, *19*), meaning that the response cannot trivially be connected to specific molecular motifs. Probing the H─O─H bending vibration is therefore often considered a more favorable approach for elucidating the molecular structure at the interface as the bending vibration is a single mode largely localized to each molecule that approximately aligns with the molecular dipole and has minimal contributions from coupling (*3*, *20*, *21*). This hence presents a more direct and simpler route to elucidate the molecular structure at the air-water interface.

A very detailed view of the anisotropic structure at the air-water interface can thus be gained by analyzing the depth/layer-dependent anisotropic response of the bending mode that is accessible from the depth-dependent second-order susceptibility, $\chi^{\left(2\right)}\left(z\right)$. This, however, requires the spectra to be of purely electric dipolar origin [i.e., containing only the interfacial dipolar (ID) signal]. Although this is readily obtained for the calculated spectra from simulations, the extent to which the experimental SFG signals are solely of electric dipolar origin is less clear and a long-standing question in second-order spectroscopy (*22*, *23*). Beyond the EDA, electric quadrupolar and magnetic dipolar signals [combined here into a generalized quadrupolar contribution, as per the conventions of Morita (*4*)] can also contribute to the obtained spectra. Unlike electric dipolar responses, these signals are insensitive to molecular orientation and thus do not report on the structural anisotropy. Therefore, the presence of any nonnegligible quadrupolar signals can obscure the desired structural information. It is thus critical that the presence of these signals is either ruled out or their contributions removed.

**Fig. 1.**
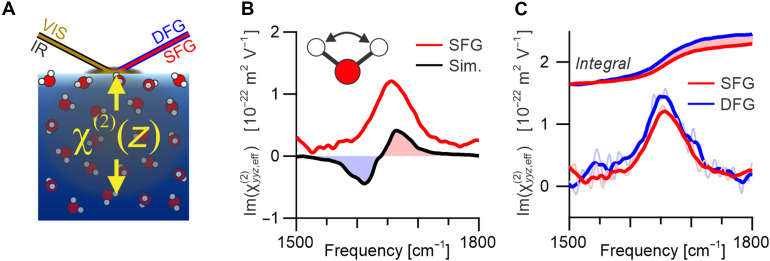
Second-order bending spectra of the air-water interface. (**A**) Schematic of the depth-resolved second-order spectroscopy from the air-water interface showing the IR + visible → SFG + DFG. In the presented colinear geometry, the SFG and DFG emerge at the same output angle as the reflected incident beams, which are omitted for clarity. (**B**) Comparison of the experimental (SFG) spectrum of the H─O─H bending mode (red) with the ID-only spectrum calculated from MD simulations (black). (**C**) SFG (red) and DFG (blue) spectra of the H─O─H bending mode of the pure air-water interface, shown both as the obtained spectra (lighter colors) and with smoothed line shapes. Also shown are the cumulative spectral integration traces.

In this work, we probe the water bending mode using our recently developed spectroscopic tool which combines phase-resolved SFG with its analogous and simultaneously generated difference-frequency (DFG) response (see schematic in Fig. 1A) (*24*, *25*). Because of the characteristic signatures of anisotropic (dipolar) and isotropic (quadrupolar) signals in the SFG and DFG responses, this technique can accurately separate them (*26*, *27*). The results are therefore divided into two parts. In the first part, we apply the SFG-DFG technique to the water bending mode spectra of both the pure air-water interface and those with surface charges to isolate the ID response. This interfacial spectrum is then quantitatively compared to the prediction from MD simulations. Thereafter, in the second part, we analyze the details of its line shape and depth dependence. This analysis reveals that the conventional model for interpreting SFG spectra in terms of the preferential molecular orientation (“pointing up/down” molecular dipoles) is utterly insufficient for the air-water interface. Through a revised model, we show that the anisotropic water structure is defined not only by a restricted and layer-dependent molecular tilt angle but also by a highly restricted and correlated molecular twist (rotation around the molecular dipole). These findings provide a refined and more detailed picture of the anisotropic structure at the air-water interface and, by further analyzing the depth-dependent frequency shifts from these structural motifs, uncover an anomalous character to the interfacial H-bonding.

## RESULTS AND DISCUSSION

### Assessing the origin of the H─O─H bending response

Although traditionally assumed, the dominant role of the ID contribution in SFG spectra has recently been questioned for the case of the water bending mode at the air-water interface. Clear evidence for this comes from the notable disagreement between the measured total and calculated ID-only line shapes, as shown in Fig. 1B (only showing the imaginary parts, i.e., the absorptive line shapes). Specifically, the simulated ID-only spectrum shows a “dip-peak” line shape whereas the experimentally obtained response shows only a single positive resonance (*20*, *28*, *29*). Although initial SFG intensity measurements seemed to reproduce the simulated bipolar line shape, this was subsequently shown to source from interference with the large nonresonant contribution (*28*–*31*). More accurate phase-sensitive measurements (which are not subjected to such interference effects) instead reveal the single positive resonance at ~1650 cm^−1^ (Fig. 1B) (*32*, *33*). This substantial discrepancy indicates that either the experimental spectrum contains considerable quadrupolar contributions or the simulations are inaccurate, and with it potentially also our current view of the interfacial water structure altogether.

The first experimental indications for the significance of quadrupolar contributions to the bending mode spectrum came from Tahara and co-workers who performed the initial phase-sensitive measurement of the air-water interface in this frequency region (*32*). Their results from studies both with and without the addition of salt indicated that the apparent single positive resonance is dominated by quadrupolar signals. A more rigorous analysis was later performed on charged aqueous interfaces. These present important field-induced molecular alignment and thus an enhanced ID response that would take the form of a single resonant band that flips sign upon charge inversion, unlike any quadrupolar signals. Although different initial measurements yielded contradicting results and produced considerable controversy about the mechanistic origin of the observed bending signals (*31*, *34*–*36*), Bakker and co-workers (*33*) recently demonstrated that both a charge-independent and charge-dependent contribution must be present in the measured spectra. This result confirms the significance of quadrupolar signals in the bending region but clearly also demonstrates that ID signals can become relevant when preferential molecular orientation is induced by surface charges. These experimental results suggest the presence of a dominant quadrupolar contribution to the pure air-water interface spectrum and hence offers an explanation for the substantial disagreement between the experimental and simulated spectra. Recent theoretical calculations have shown a bulk-like quadrupolar response to be the dominant contribution in the bending response (*27*, *32*). Nevertheless, the experimental ID spectrum from the air-water interface that is required for a reliable structural analysis remains elusive. For a more detailed description of the different quadrupolar contributions and their relevance to the bending mode spectrum, see the Supplementary Materials.

The exact origin of the SFG response can be assessed using the SFG-DFG technique (*24*–*26*, *37*). SFG and DFG contributions that originate from structural anisotropy (dipolar signals) have equal intrinsic (local) responses. However, because of the different wave vector mismatches for SFG and DFG (opposite signs and different amplitudes), integration of the intrinsic (local) responses over depth leads to different phase shifts and amplitude scaling factors. The induced phase difference between the obtained SFG and DFG spectra amounts to ~2°/nm in depth, making the technique a very sensitive tool for analyzing the thickness of the structural anisotropy (*37*). For signal contributions from structurally isotropic regions (quadrupolar), the situation is different. Here, the corresponding intrinsic (local) responses in SFG and DFG are no longer equivalent, ultimately leading to distinct amplitudes but with no phase difference in the overall response (*26*, *27*). On the basis of these characteristics, combining the phase difference and amplitude ratio of the SFG and DFG spectra allows for the separation of the anisotropic and isotropic contributions.

The SFG and DFG bending responses (imaginary parts) of the pure air-water interface are depicted in Fig. 1C. By comparing the two spectra, it is first clear that they exhibit little if any phase difference, which would present as oppositely distorted spectral line shapes because both exhibit a single positive resonance with an almost symmetric line shape centered at ~1656 cm^−1^, as previously reported (*33*). This observation aligns with a previous work showing that the anisotropic structure at the air-water interface is contained within an exceptionally small region of ~6 to 8 Å (*26*). On further inspection, however, it is also clear that the DFG response has a larger amplitude. This difference is emphasized in the spectral integral traces also shown in Fig. 1C. The observation of little phase difference but a distinct amplitude offset is therefore indicative of a large isotropic quadrupolar contribution, reaffirming the importance of quadrupolar contributions to the bending mode. Furthermore, the amplitude difference between SFG and DFG indicates that the quadrupolar contribution arises from a bulk response and therefore should present a bulk-like spectrum. Overall, despite slight differences in the spectra predicted by theory with those measured in experiments, both agree that the bending mode spectrum should have a dominant quadrupolar component that resembles bulk water (see the Supplementary Materials for a more detailed discussion on this point).

### The dipolar response from charged interfaces

The presence of this large bulk-like contribution to the pure air-water interface spectrum clearly hinders a detailed analysis of the anisotropic interfacial water structure at this point because it buries the desired interfacial contribution. This raises the important question how large the dipolar response is, and can it be retrieved from the total line shape? To address these questions, we move to charged aqueous interfaces. Measuring the SFG and DFG responses from these systems has a distinct advantage for obtaining a better understanding of the pure air-water interface. Specifically, the net surface charge generates a static electric field which propagates away from the interface and creates a torque on the molecular dipoles, inducing anisotropy through preferential molecular orientation (*37*–*41*). This generates a much larger dipolar response than for pure air-water. Furthermore, its sign can be inverted by changing the sign of the surface charges. This makes it possible to isolate the dipolar contribution from the overall response and assess its line shape. In addition, the field-induced anisotropy is also present several nanometers away from the interface, and the extent of the reorientation in these regions is small such that the local intermolecular environments are largely maintained (*42*). This means that the contributions from these regions represent a bulk-like spectrum and should thus have the same line shape as the quadrupolar response. This holds despite their different symmetry rules as they are both second-order responses and arise from only a single vibrational mode. If this bulk-like contribution to the dipolar response can be isolated from the overall charged interface spectra, its line shape can then be directly used to remove the quadrupolar contribution to the pure air-water spectrum and reveal the elusive dipolar response.

**Fig. 2.**
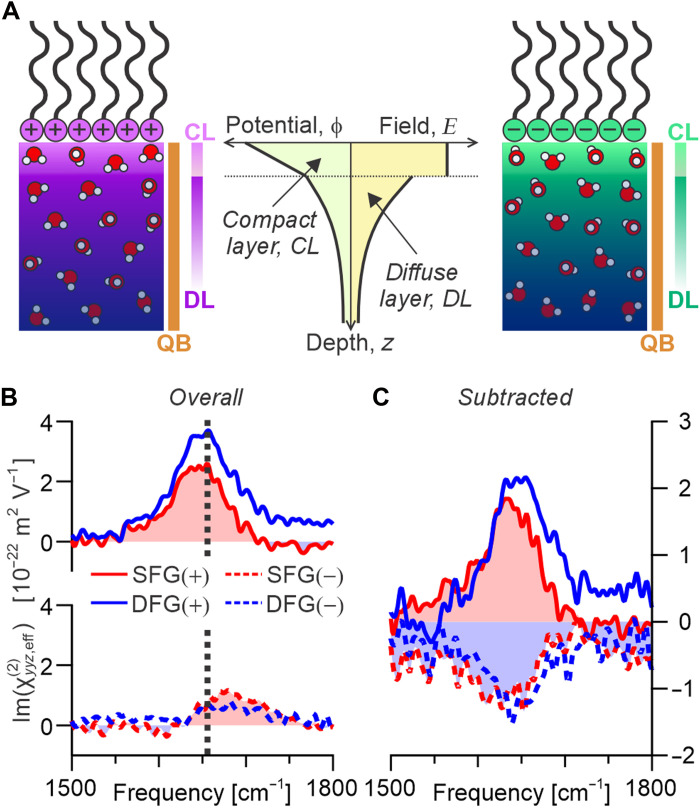
Water bending spectra at charged interfaces. (**A**) Schematic representation of the GCS model for charged aqueous interfaces, showing the evolution of electrostatic potential and electric field with depth, indicating the two distinct dielectric regions: the CL and DL. Also shown are schematics of the anisotropic water structure for both positive and negative charged surfaces as well as bar representations of the different contributions to the SFG (and DFG) responses [including the bulk quadrupolar contribution (QB)]. (**B**) SFG and DFG spectra for the positive (solid) and negative (dashed) surfactants, with the dashed black line indicating the position of the peak frequency in the pure air-water spectrum. (**C**) Same surfactant spectra shown in (B) having subtracted the pure air-water interface spectrum shown in Fig. 1D.

Following the description of the depth-dependent electrostatics at charged interfaces within the Gouy-Chapman-Stern (GCS) model (shown schematically in Fig. 2A), the anisotropic structure is described by two distinct layer contributions: the compact layer (CL), which represents the thin layer of anisotropy induced by the presence of the interface, and the diffuse layer (DL), which only gives rise to a field-induced response but can extend tens of nanometers away from the interface under low-salinity conditions (*43*, *44*). It is thus the DL contribution that represents the aforementioned desirable “bulk-like” response. As the CL and DL contributions to the dipolar signal arise from different depths, SFG-DFG can fully resolve their contributions and isolate their spectral line shapes (*37*).

The measured spectra for interfaces with both positively and negatively charged surfactants are shown in Fig. 2B. For this, we use the surfactants dihexadecyldimethylammonium bromide (DHAB) and dihexadecyl phosphate (DHP), which are both highly insoluble (thus allowing for high surface coverage without substantial bulk concentrations) and free from any resonances that could interfere with the water bending response. On initial inspection, the spectra from both charges exhibit positive bands. The absence of a clear sign flip in the two responses immediately shows that the bulk quadrupolar signal must still be a large contribution to the spectra. However, further comparison shows that, although the peaks in the spectra do not have opposite signs, the positive surfactant yields spectra that are enhanced in amplitude and slightly red-shifted (compared to pure air-water; see dashed black line) and the negative surfactant yields spectra that have diminished amplitudes and slight blue shifts. This indicates that the spectra contain both a charge-dependent (dipolar) and charge-independent (quadrupolar) contribution, as previously reported (*33*).

An approximate method of separating the charge-dependent dipolar contribution from the charge-independent quadrupolar contribution is to simply subtract the spectrum from the pure air-water interface, which is shown above to be dominated by the same bulk-like quadrupolar response. This leaves only the dipolar contributions from the CL and DL, as well as potentially a minor contribution from the dipolar air-water spectrum introduced from the subtraction. It is clear from comparing these spectra, shown in Fig. 2C, that they now display the expected sign flip upon charge inversion, confirming their dipolar origin. Beyond this, comparing the corresponding SFG and DFG spectra shows that they are clearly phase shifted, presenting different line shapes. This highlights the substantial anisotropic depth in these systems, with the DL contributions extending over 100 nm away from the interface (given a salinity of <10^−5^ M). Overall, this conclusively demonstrates that the bending spectra at charged interfaces do contain a field-dependent dipolar response and a field-independent quadrupolar response, with both being similar in size.

### Extracting the dipolar response of the air-water Interface

To precisely isolate the desired pure bulk-like DL spectrum, we combine all four charged interface spectra without subtracting the pure air-water response (i.e., the “overall” spectra from Fig. 2B). Full details of this extraction are given in the Supplementary Materials. Briefly, the difference spectrum between SFG and DFG for each charge removes their CL contributions, and then the subtraction of these difference spectra for the two charges eliminates the quadrupolar contributions while constructively combining the DL responses (which have opposite signs for the two charges). Last, this isolated DL contribution is corrected for its depth-related phase shift (*42*, *45*).

**Fig. 3.**
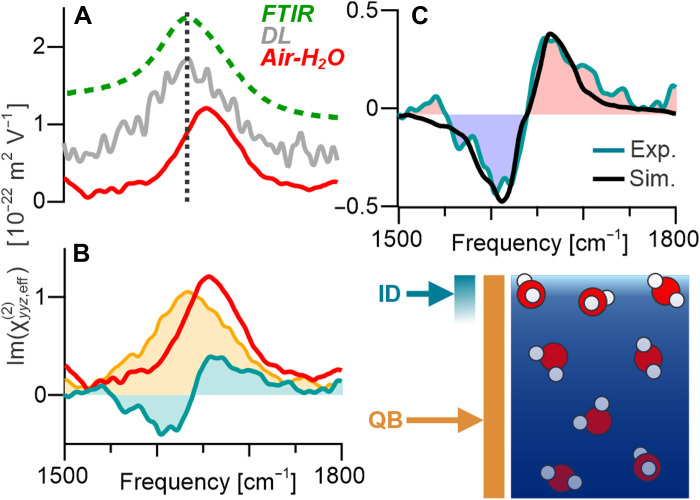
Spectral decomposition of bending response. (**A**) Spectra for the pure air-water interface (SFG; red) and DL (gray) from the charged interfaces, extracted on the basis of the GCS model, along with the linear FTIR spectrum (dashed green) for comparison. The three spectra have been offset for clarity. (**B**) Pure air-water interface SFG spectrum (red) along with its decomposition into an isotropic (bulk) quadrupolar contribution (orange) and an anisotropic (interfacial) dipolar contribution (blue). The spatial origins of these two sources are also schematically indicated. (**C**) Extracted dipolar contribution to the pure air-water interface spectrum overlapping with that obtained through MD simulations.

The obtained DL spectrum is shown in Fig. 3A (gray trace). The spectrum is shown alongside the pure air-water interface SFG spectrum (red; also shown in Fig. 1, A and D) and the Fourier transform infrared (FTIR) spectrum of pure water, representing an approximation for the effective bulk $\chi^{\left(1\right)}$ response (dashed green). On comparison, the DL spectrum matches exceptionally well with the FTIR spectrum, with both presenting a single band centered at ~1637 cm^−1^ with a full width at half maximum (FWHM) of ~90 cm^−1^. The only noticeable difference exists for the low frequencies that are known to contain additional intensity in FTIR spectra owing to the first overtone of the low-frequency libration modes (*46*). This agreement validates the above assumption that the DL spectrum is a bulk-like spectrum. By contrast, if we compare the spectrum from the pure air-water interface to the bulk-like spectra of either the DL or FTIR, there are clear line shape differences, particularly in peak frequency (1656 cf. 1637 cm^−1^). As the DL and quadrupolar responses should have the same line shape, this discrepancy hence must arise from the ID response in the pure air-water spectrum.

By subtracting the bulk line shape (DL spectrum) from the overall spectrum of the pure air-water interface, the quadrupolar contribution is removed, uncovering a residual component with a defined “dip-peak” spectrum (Fig. 3B; see the Supplementary Materials for more details on this subtraction). A comparison of this component to the ID-only prediction from MD simulations (Fig. 3C) shows perfect agreement in both line shape and absolute amplitude, therefore conclusively confirming that this spectral feature is the elusive ID contribution. Such verification of the predictions of MD simulations are crucial for obtaining confidence in their structural findings.

### Elucidating the depth-dependent interfacial water structure

**Fig. 4.**
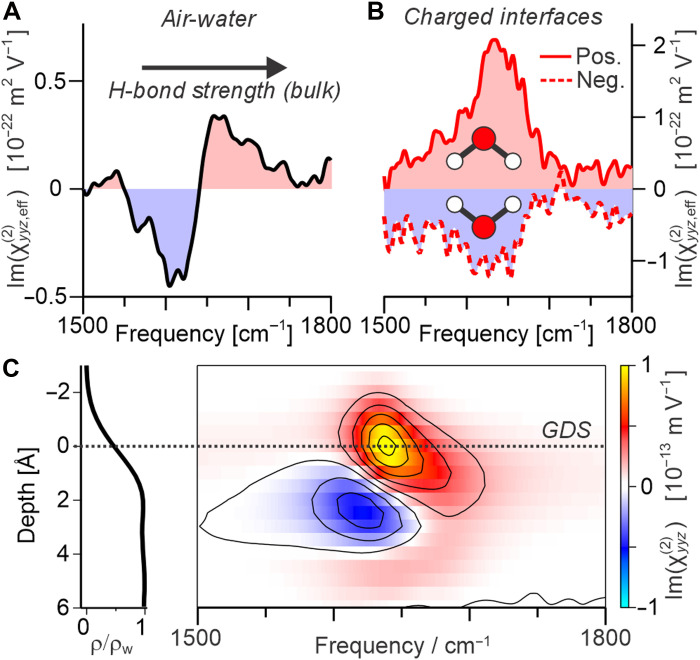
Analysis of depth-dependent response. (**A**) Extracted interfacial (dipolar) spectrum for the pure air-water interface, as shown in Fig. 3C. (**B**) SFG spectra for both surfactants having subtracted the pure air-water interface spectra, as shown in Fig. 2C, indicating the average preference for the molecular dipole pointing down (positive surfactants) and up (negative surfactants). (**C**) Depth-dependent ID-only bending response calculated from MD simulations, also showing the depth-dependent density profile and the GDS, defining zero depth.

With the ID response isolated and the theoretical predictions experimentally verified, we can now confidently analyze the structural implications of the obtained spectrum. The “dip-peak” line shape is characteristic of two overlapping contributions: a red-shifted dip and a blue-shifted peak. Each of these spectral features then contains two important pieces of contrasting information, their peak frequency and their sign, which relate to the intermolecular connectivity and molecular orientation, respectively. To analyze the peak frequency, one typically compares the known frequency shifts due to H-bonding in bulk water, where a red shift in the bending mode indicates weaker H-bonding (in direct contrast to the O─H stretch). For the sign of the response, one typically compares them to the sign obtained for the dipolar contribution from charged interfaces. As these responses arise from field-induced reorientation, the peak observed for the positively charged interface should be dominated by molecules with their dipoles pointing “down” (toward bulk water) and the dip observed for the negatively charged interface from molecules with their dipoles pointing “up” (toward the air phase), as indicated in Fig. 4B. This would therefore suggest that the red-shifted dip for air-water arises from “pointing up” molecules with weaker H-bonding and the blue-shifted peak from “pointing down” molecules that are more strongly interconnected (*28*, *29*, *47*). This interpretation is fully compatible with the expectations and current “picture” of the interface as one would expect molecules “pointing down” to have greater access to H-bonding than those pointing toward air.

To further analyze the interfacial structure, one can then turn to the depth dependence of the bending response predicted by MD simulations. This is shown in Fig. 4C, which plots the two-dimensional (2D) ID-only second-order susceptibility, $\chi_{\mathrm{yyz}}^{\left(2\right)}\left(z\right)$. From this, one can clearly identify both the positive response at higher frequencies and the negative response at lower frequencies. These appear, however, to be spatially separated, i.e., arising predominantly from different depths. In particular, the blue-shifted positive contribution clearly sources from closer to the air phase. Following the interpretation of the spectral line shape discussed above, this would therefore suggest that the topmost water molecules are predominantly “pointing down” and more strongly H-bonded than those located below which predominantly “point up.” This picture clearly no longer fits with our expectations of the topmost water having dangling bonds and being more weakly H-bonded or equally with previous analysis of the SFG stretching mode spectra (*26*, *37*, *48*–*50*) or even calculations of the preferential molecular orientation at the interface (*51*).

**Fig. 5.**
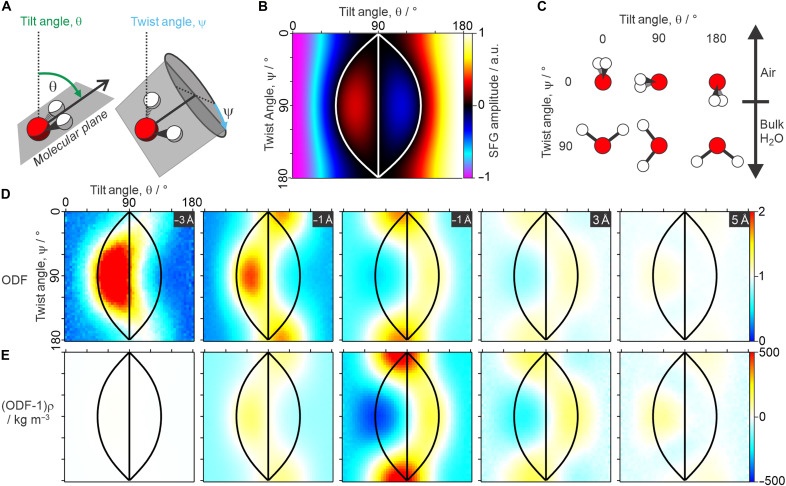
Tilt/twist orientational analysis. (**A**) Schematic of the molecular tilt and twist angles, showing the defined molecular plane that contains all three atoms in H_2_O. (**B**) Calculated SFG amplitudes for the bending mode (see the Supplementary Materials for details). a.u., arbitrary units. (**C**) Representation of the molecular orientation at the interface for specific combinations of tilt and twist. (**D**) Calculated 3D ODF (tilt versus twist versus depth) from MD simulations, shown at specific depths through the interface, also shown in (**E**) having normalized for the molecular density across the interface.

The above analysis of both the experimental spectra and its calculated depth dependence clearly shows that the typical way of interpreting the bending mode spectrum in terms of “pointing up” and “pointing down” molecules must be incorrect and should be substantially revised. Starting from first principles, the amplitude of the SFG response from an individual water molecule can be calculated as a function of its 3D molecular orientation (i.e., neglecting any frequency dependence associated with its interconnectivity). The spatial mapping of the molecular coordinates onto the interface is often described by mutually dependent angles (*52*), e.g., its two O─H bonds (*53*, *54*); however, it is often far more intuitive to describe this mapping using the three independent Euler angles: tilt, twist, and azimuth. In this description, the tilt angle is defined as the angle between the molecular dipole and the surface normal, the twist angle is the amount of rotation around the dipole axis, and the azimuth is simply associated with the direction of the in-plane projection of the molecular dipole, which is isotropically distributed at such liquid interfaces. A schematic of the tilt and twist angles is shown in Fig. 5A. By combining this orientational projection with the molecular symmetry properties and a simple geometric description of the bending vibration (see the Supplementary Materials for details), Fig. 5B then shows the calculated relative SFG amplitude of a single water molecule as a function of both the tilt and twist angles. For better clarity, Fig. 5C gives schematics of the molecular orientation at the interface for selected combinations of the two angles.

The theoretical plot of SFG amplitudes in Fig. 5B shows that both the magnitude and sign dependence of the bending response for different molecular orientations is far from simple. Specifically, although it is loosely true that molecules “pointing up” (with tilt angles < 90°) yield negative responses and molecules “pointing down” (with tilt angles > 90°) yield positive responses, there are clear regions in the plot that show the opposite, with this highlighted by a contour line at zero amplitude (shown in white). Molecules “pointing up” can yield positive or negative responses, depending on their twist angle. This hence raises the question whether the aforementioned contradiction between the depth-dependent response and the conventional interpretation of the spectral line shape arises due to the observed signals sourcing from molecules with orientations in these sign-flip regions. In other words, is the twist angle at the interface orientationally restricted and correlated to the tilt angle?

The answer to this question lies in the full 3D orientational distribution function (ODF) at the interface, i.e., tilt versus twist versus depth (*52*). This was hence calculated from the MD simulations and is presented in Fig. 5D using the Euler description. Specifically, Fig. 5D shows the tilt versus twist distribution at 2-Å steps through the interface, where regions displaying yellow or red (i.e., ODF values > 1) indicate molecular orientations that are populated more than they would be for an isotropic distribution, and those in cyan and blue (ODF values < 1) represent the less populated orientations with respect to an isotropic distribution. On initial inspection, the ODF above the interface (toward the air phase), at −3 Å, does show that the preferential molecular orientation at this depth lies almost solely in the calculated sign-flip region (indicated by the same contour lines shown in white in Fig. 5B being repeated here in black). Specifically, it shows a clear preference for water molecules with their dipoles pointing slightly “up,” but with a 90° twist, meaning that they are effectively tilted in the molecular plane directing one O─H bond toward the air phase, i.e., the “free O─H groups” known from previous measurements in the stretching region (*26*, *55*) (see Fig. 5, A and C). Below this, at −1 Å, the same molecular orientation is still preferential, but with a diminished enhancement, and a second orientation becomes favorable, specifically molecules slightly pointing “down” with 0°/180° twist, i.e., with both O─H bonds being almost parallel to the interface. Thereafter, on the other side of the Gibbs dividing surface (GDS; which defines *z* = 0), this is then essentially mirrored for +1 and +3 Å, with the preference being for “pointing up” with 0° twist and “pointing down” with 90° twist. Last, for +5 Å, it appears to flip again, showing a similar distribution as for −1 Å, but with markedly lower population enhancement due to the increasing tendency toward an isotropic distribution. Overall, this clearly demonstrates that the twist angle is not only highly restricted at the interface but also strongly correlated to the tilt angle and that both are strongly varying as a function of depth. Although this structural information is likely also contained within the results of previous MD simulations (*51*–*54*, *56*), the insights on these tilt-twist motifs only become apparent due to using the Euler representation and calculating the full 3D ODF, as presented here. Furthermore, the presented orientational insights are here directly correlated to the corresponding spectral signatures, generating a solid framework for similar studies in the future.

This depth-dependent flipping in the preferential molecular orientation (both tilt and twist angles) is strongly indicative of a dominant role of the local H-bond network on the orientational distribution. This is suggested as a 90° change in twist corresponds precisely to flipping between the H-bond donor plane (same as the molecular plane; see Fig. 5A) and the plane of the H-bond acceptors (O lone pairs). Therefore, it seems that the highly directional and approximately tetrahedrally oriented nature of the H-bonding is imprinted on the molecular orientations directly at the interface. Although these macroscopic orientational correlations are not seen in the bulk due to its isotropic nature, they clearly appear at the interface. The resulting pronounced layering must therefore originate from a specific preferential orientation in the topmost layer that is induced by the macroscopic phase boundary. The orientation in this topmost layer then restricts the orientational distribution in the subsequent layers due to their thermodynamic preference for maximizing their interconnectivity.

It is important to note that, although the regions toward the air phase present the strongest preferential orientation, the density of water molecules closer to the air phase is much smaller than on the other side of the GDS. This means that, considering their density, these regions contribute essentially nothing to the overall SFG signals. This effect is accounted for in Fig. 5E, which scales the ODF for density (but now with a different normalization such that 0 corresponds to an isotropic distribution). As expected, the density-scaled ODF at −3 Å shows negligible contributions. Below this, however, there are clear contributions from the abovementioned orientations spanning the presented ~6- to 8-Å range in depth.

**Fig. 6.**
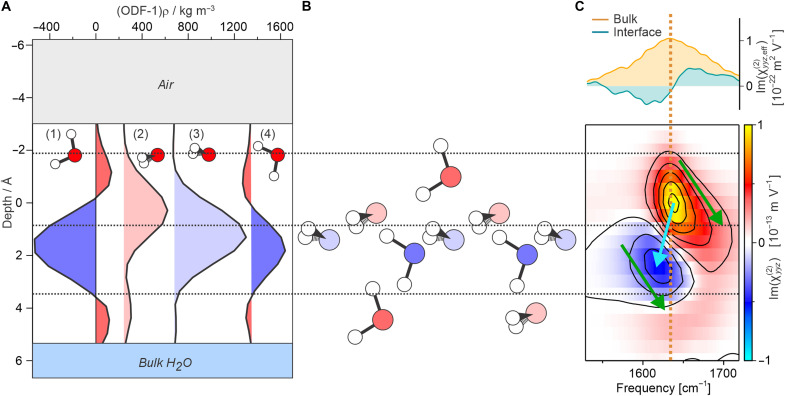
Analysis of depth-dependent populations. (**A**) Depth dependence of specific orientations that were selected from the observed population peaks in the density-normalized ODF shown in Fig. 5E. (**B**) Schematic representation of the preferential (anisotropic) orientation at the air-water interface. The fill colors of the traces in (A) and molecules in (B) approximately represent the amplitude of their SFG contribution. (**C**) Depth-dependent second-order susceptibility, $\chi_{\mathrm{yyz}}^{\left(2\right)}\left(z\right)$, shown with the experimentally determined interfacial spectrum, as well as the bulk-like spectrum for comparison. The green arrows highlight the depth-dependent blue shift for each of the positive and negative responses, whereas the blue arrow highlights the red shift between the different sign responses.

As discussed above, analysis of the ODF shows that there are essentially four distinct classes of preferential orientations at the air-water interface (as defined by the distinct peaks in populations): (1) pointing slightly “up” with 90° twist, (2) pointing slightly “down” with 0° twist, (3) pointing slightly “up” with 0° twist, and (4) pointing slightly “down” with 90° twist. As such, the anisotropic structure in terms of molecular orientation can be well described from the depth-dependent populations of these four structural motifs, which are shown in Fig. 6A through their depth-dependent contributions to the density-scaled ODF. The fill color of these traces approximately represents their contributed SFG amplitude, estimated from the calculated response shown in Fig. 5B (where diminished populations compared to an isotropic distribution are assigned the opposite sign response). Figure 6B then shows a schematic representation of this anisotropic structure at the air-water interface, again where the fill color of the molecules highlights their contributed SFG amplitude. These approximate amplitudes (as well as those in Fig. 6A) can be compared to the depth-dependent $\chi^{\left(2\right)}\left(z\right)$ calculated from simulations shown in Fig. 6C (just as in Fig. 4C). This comparison shows overall excellent agreement in terms of the depth-dependent sign of the response, with structural motifs (1) and (2) dominating the topmost region and both yielding a positive response and motifs (3) and (4) dominating the region below, both yielding negative signals. Last, the weak positive contribution from ~3 to 5 Å arises from a slight preference for motifs (1) and (2) again.

At first sight, the 3D “picture” of the anisotropic interfacial orientations in Fig. 6B indicates that the structural motifs (2) and (3) that dominate the interfacial anisotropy from ~0 to 2 Å could be part of a macroscopic “buckled” 2D water network, as previously suggested from simulations (*51*, *57*, *58*). However, for a clear assessment of any network structure, the precise intermolecular connectivity would have to be determined. Such an analysis is highly involved and thus requires substantial further work. Nevertheless, some insights can be obtained through the apparent depth-dependent frequency shifts in the bending response. On inspection of Fig. 6C, there are two clear types of depth-dependent frequency shifts: (i) a distinct blue shift of both features indicated by the green arrows and (ii) a general red shift of the negative feature cf. the positive feature, as indicated by the blue arrow. Although the gradual depth-dependent blue shift in both features could be a manifestation of the increasing H-bonding strength with depth, the overall displacement of the negative response to lower frequencies, despite its larger depth, strongly deviates from this. The different shifts of the bend and stretch water bands when going from vapor to the liquid phase (red for stretch and blue for bend) was recently explained in terms of the competition of the bond elongation when entering the liquid phase, which induces a red shift, and frequency-dependent friction effects, which induce a blue shift (*59*). A similar competition of bond elongation and dissipation is expected when moving through the interface from vapor to bulk liquid. A comparison of the absolute frequencies of these features to the frequency of the bulk response (indicated by the dashed orange line in Fig. 6C) actually shows that topmost layer seems to display a more bulk-like spectrum than the layers beneath. Overall, therefore, analysis of the depth dependence of the vibrational frequency shows that the anisotropic interconnectivity at the air-water interface and its connection with the vibrational bending frequency is far from simple and thus requires further theoretical investigation. The above observations, however, highlight that either the interconnectivity is far from a monotonic function with depth and/or that the factors governing the bending response at the interface, i.e., the different aspects of the H-bonding network, vastly differ from the bulk.

In summary, by applying our recently developed depth-resolved second-order spectroscopy to study the H─O─H bending mode, we have separated out a substantial bulk-like quadrupolar contribution to the air-water interface spectrum and extracted the previously unobserved ID response. This is shown to quantitatively match the calculated spectrum from MD simulations, thus finally verifying the theoretical predictions and allowing a more in-depth investigation into the interfacial structure. Through an analysis of the spectral line shape of the extracted interfacial response, as well as its calculated depth dependence, we then demonstrate that the conventional understanding and interpretation of the water spectrum in terms of “pointing up” and “pointing down” molecular dipoles not only is incorrect but also misses out on a crucial part of the preferential orientation at the interface, namely, the twist angle. From the full calculated orientational distribution, we clearly demonstrate that both the tilt and twist are highly restricted, correlated to one another, and show a pronounced layer/depth dependence. Specifically, we highlight that the anisotropic structure and its resulting SFG amplitudes can be well described by four distinct classes of molecular orientation, with dipoles pointing either slightly “up” or slightly “down” and twist angles close to either 0° or 90°. Last, by analyzing the depth dependence of the vibrational frequencies, we show that the interconnectivity at the interface is highly complex and either not well described by a simple combination of orientation and monotonic depth dependence to the H-bonding or that it shows a non-bulk-like connection between vibrational frequency and interconnectivity. In either case, this highlights the anomalous nature of the interfacial H-bond network.

Overall, these findings give a refined and detailed picture of the air-water interface, with specific structural motifs defining a pronounced layered structure. However, the interface still has a substantial proportion of isotropically distributed water—these structural motifs simply dominate and define its anisotropy. Nevertheless, presenting a distinct layered structure with clear preferential orientations shows that the interfacial water has fewer orientational degrees of freedom than in the bulk, which has important implications for interfacial processes. Specifically, the dynamics of the H-bond fluctuations will be altered, which will have a crucial impact on processes such as proton hopping, transport processes, and chemical reactions. Although these structural observations have been shown here for the pure air-water interface, this serves as a useful model for a wide range of aqueous interfaces because it can be expected that interfacial water adopts similar conformational restrictions across many interfaces.

## MATERIALS AND METHODS

### Sample preparation

The spectroscopic measurements of the aqueous interfaces were performed on both H_2_O [Milli-Q; 18.2 MΩ cm, <3 parts per billion (ppb) total organic carbon (TOC)] and D_2_O (VWR Chemicals, 99.9% D) contained in custom-made polytetrafluoroethylene (PTFE) troughs that were cleaned overnight with Piranha solution (3:1 sulfuric acid to 30% hydrogen peroxide solution) and subsequently thoroughly rinsed with ultrapure water. Warning: Piranha solution is highly corrosive and an extremely powerful oxidizer. Great care must be taken with its preparation and use.

For the measurements at charged interfaces, the two surfactants, DHP and DHAB, were prepared as a solution (1 mg ml^−1^) in chloroform and deposited dropwise onto the water surface in the PTFE troughs until surface saturation was reached. This procedure ensured a consistent well-packed monolayer film of sufficient density as to minimize any effects from Bénard-Marangoni convection (*60*).

### Spectral acquisition

The details of the SFG and DFG interferometer used to measure the spectra shown here can be found elsewhere (*61*). Briefly, the 7-W 1-kHz 800-nm output from a Ti:sapphire laser (Astrella, Coherent) is fed into two independent optical parametric amplifiers (TOPAS Prime, Light Conversion), the first being used to generate mid-IR through DFG and the second producing a signal beam that is subsequently frequency doubled to produce a tunable visible upconversion. The IR output is split, with one part being colinearly overlapped with the visible in *z*-cut quartz to generate local oscillator (LO) references that are (along with the visible) subsequently colinearly overlapped with the second part. This generates a single colinear beam that is directed to the sample at 70° incidence angle to produce the signal SFG and DFG. The reflected beam is then filtered and collected on single-channel detectors, implementing balanced detection. Furthermore, for greater phase accuracy, the sample scans are recorded alongside a *z*-cut quartz reference through shot-to-shot referencing.

The acquired spectra were recorded in the time domain from −400 to 5000 fs in 0.8-fs steps to ensure sufficient spectral resolution. The presented spectra represent coaverages across multiple individual scans. Specifically, the pure air-water interface represents the average of 62,000 spectra, the DHAB measurements across 73,000 spectra, and the DHP across 79,000 spectra. During the measurements, the entire optical path was purged with dry, CO_2_-scrubbed air to minimize atmospheric absorption. In addition, to account for any changes in optical path due to sample evaporation, the sample height was corrected during each measurement using an automated stage.

### Data treatment

To correct the spectra for amplitude and phase, they were referenced using a transfer function measurement of *z*-cut quartz recorded from −400 to 3000 fs in 0.8-fs steps, taking the response from quartz to have a phase of ±90° as a close approximation, neglecting any surface effects from the quartz (*62*). For a more accurate phase correction, the spectra were further corrected using the SFG and DFG responses of the carbonyl stretch from a dipalmitoylphosphatidylcholine monolayer on fused silica (averaged over 18,000 scans), which is highly surface localized and thus should result in a minute phase difference (*24*–*26*). The resulting amplitude- and phase-corrected spectra were then corrected for the nonresonant response by subtracting analogous measurements from D_2_O.

## Supplementary Materials

### This PDF file includes:

Supplementary Text Figs. S1 to S4 References
