# Supplementary material for: The importance of layer-dependent molecular twisting for the structural anisotropy of interfacial water
Source: Sci Adv. 2026 Apr 22;12(17):eadz5505. doi: 10.1126/sciadv.adz5505 (PMC13101875; doi:10.1126/sciadv.adz5505)
Supplement: Supplementary file 1 — Supplementary Text Figs. S1 to S4 References [file sciadv.adz5505_sm.pdf]

Science Advances
AAAS

Supplementary Materials for

The importance of layer-dependent molecular twisting for the structural anisotropy of interfacial water

Alexander P. Fellows *et al.*

Corresponding author: Martin Thämer, thaemer@fhi-berlin.mpg.de

Sci. Adv. **12**, eadz5505 (2026)
DOI: 10.1126/sciadv.adz5505

This PDF file includes:

Supplementary Text
Figs. S1 to S4
References

Calculation of Spatially Resolved $\chi^{(2)}$ from Molecular Dynamics Simulations

The theoretical prediction of the spatially resolved electric dipolar contribution to the second-order susceptibility was calculated from *ab initio*-parameterized molecular dynamics simulations, full details of which can be found elsewhere.(*26, 27*) From this, the 2D frequency vs. depth map of the yyz component, $\chi^{(2)}_{yyz}(z)$, was extracted in absolute units. The corresponding spectrum of the effective second-order susceptibility, $\chi^{(2)}_{yyz,eff}$, was then calculated by integrating the response over depth.

For the analysis of the depth-dependence of the response, only the intramolecular component of the purely electric dipolar response function was used. With the overall response function, $s^{(2)}_{ijk}$, being defined as in Eq. S1, relating to the total systems dipole moment, P_i, we therefore neglect the intermolecular terms and only consider the dipole moments of the individual molecules, μ_i^n, as in Eq. S2,

$$s^{(2)}_{ijk}(z,t) = \frac{-\Theta(t)}{Ak_B T\varepsilon_0}\frac{\partial}{\partial t}\sum_{n}^{N_{mol}}\langle \alpha^n_{ij}(t)\delta[z - z^n(t)]P_k(0)\rangle \qquad (S1)$$

$$s^{(2)}_{ijk}(z,t) \approx \frac{-\Theta(t)}{Ak_B T\varepsilon_0}\frac{\partial}{\partial t}\sum_{n}^{N_{mol}}\langle \alpha^n_{ij}(t)\delta[z - z^n(t)]\mu^n_k(0)\rangle \qquad (S2)$$

where, A, k_B, T, and z^n are the interfacial area, the Boltzmann constant, the temperature, and the z-position of the nth molecule, respectively. The Heaviside function is denoted as $\Theta(x)$ and the Dirac delta distribution by $\delta(x)$. The number of molecules is N_{mol}. The extraction of the effective electric dipole polarizabilities α^n_{ij} is described elsewhere.(*26*)

Isolating the purely intramolecular component of the response function has the benefit that it has a significantly better signal-to-noise ratio and can be better understood when assessing the anisotropic structure at the interface. Particularly, for comparison to the SFG amplitudes of the bending response for different molecular orientations calculated purely on theoretical grounds (shown in Figure 5b in the main text), using only the intramolecular response is a more direct comparison. In any case, the comparison between the purely intramolecular contribution to $\chi^{(2)}_{yyz}(z)$ and the total response presented in Figure S1 shows that neglecting the intermolecular component has little effect. The total response shows good qualitative overlap with the intramolecular response, reproducing the same positive and negative features in similar positions and still shows the main frequency-shifts as highted in Figure 6c and discussed in the main text.

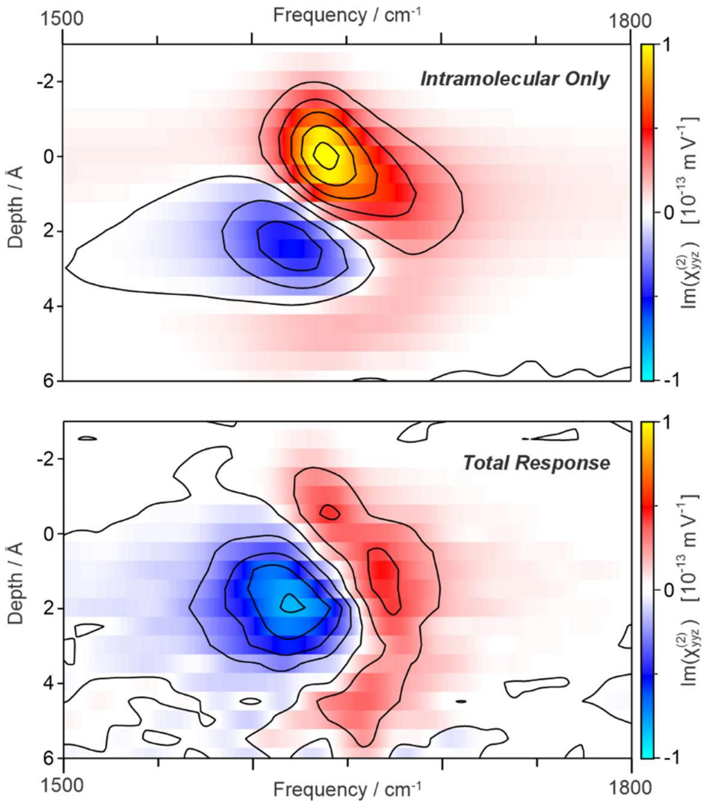

Figure S1: Comparison of the intramolecular contribution to $\chi^{(2)}_{yyz}(z)$ *and its total response.*

Finally, the simulations were performed for both H_2O and D_2O, giving two independent comparisons of the resulting dipolar spectrum to experiment. While the comparison to the calculated H_2O spectrum, in principle, directly compared the same frequency range, the minor nuclear quantum effects (smaller than those in the O-H stretch) that are not accounted for in the simulations result in the calculated spectrum being shifted by ~35 cm^{-1} to higher frequencies. Nevertheless, shifting the spectrum by this value results in exceptional overlap, as shown in Figure S2.

When comparing the experimental H_2O spectrum to the calculated D_2O spectrum, one must account for the natural frequency shift caused by the change in reduced mass. This results in an effective frequency conversion factor of $\frac{3}{\sqrt{5}}$ which, when applied to the calculated spectrum, also yields exceptional overlap to the experimental results, as shown in Figure S2.

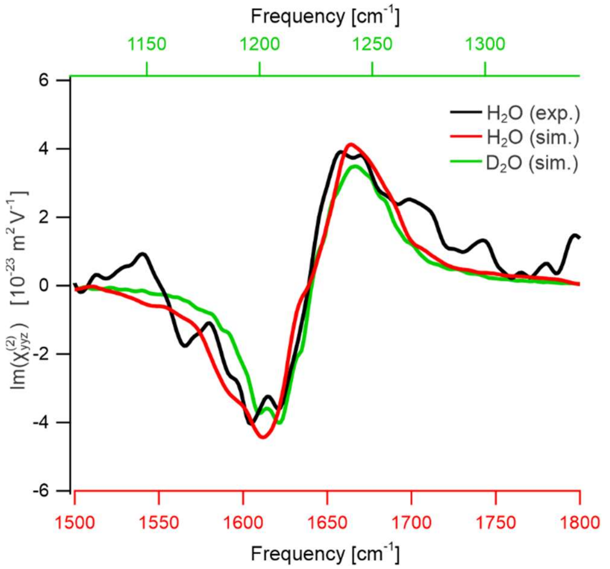

Figure S2: Comparison between the experimentally isolated ID spectrum and the predicted ID-only spectra of both H_2O and D_2O calculated from simulations. The D_2O spectrum is shown on the top axis, having calculated the corresponding frequency range based on the difference in reduced mass for the bending vibration.

Spectral Contributions, GCS Theory, and Extracting the DL Spectra

The general second-order response (SFG or DFG) from an interface probes the effective second-order susceptibility given by Eq. S3, containing both an electric dipolar (interfacial dipolar, ID) and quadrupolar (Q, taken here as combining electric quadrupolar and magnetic dipolar mechanisms, as per the conventions of Morita(*4*)) contribution.

$$\chi^{(2)}_{eff} = \chi^{(2)}_{ID} + \chi^{(2)}_{Q} \qquad (S3)$$

For the pure air-water interface, the dipolar response can be written as in Eq. S4, including its depth-dependent decay over the anisotropic decay length, z'.(*24, 25*) Upon integration, a decay length-dependent amplitude-factor and phase-factor are introduced, as shown in Eq. S5. For very small decay lengths, as is the case for pure air-water(*26*), the amplitude-factor can be taken to be z' and the phase-factor, 1, leading to Eq. S6.

$$\chi^{(2)}_{eff,ID} = \int_0^\infty \chi^{(2)}_{ID} e^{\left(i\Delta k_z - \frac{1}{z'}\right)z} dz \qquad (S4)$$

$$= \frac{1}{\sqrt{\left(\frac{1}{z'}\right)^2 + \Delta k_z^2}} \chi^{(2)}_{ID} e^{i\,\mathrm{atan}(\Delta k_z z)} \qquad (S5)$$

$$\approx z'\chi^{(2)}_{ID} \qquad (S6)$$

On the introduction of a charged surfactant at the interface, however, a static electric field is induced, meaning the ID contribution can be further broken down into a $\chi_D^{(2)}$ and $\chi_D^{(3)}$ response, as in Eq. S7, with the latter arising from depths extending much further away from the interface (ultimately controlled by the screening of the field). Based on the Gouy-Chapman-Stern (GCS) model, the ID response can be further broken down into a compact layer (CL) and diffuse layer (DL) contribution, as described by Eq. S8, with the latter containing only the field-induced term. By considering the CL to be much thinner than the coherence length and combining the $\chi_D^{(2)}$ and $\chi_D^{(3)}$ responses, the CL contribution can be represented by a single term with minimal propagation-induced phase-shift, $\chi_{CL,eff}^{(2)}$. Furthermore, applying the exponentially decaying potential in the DL allows the integral in Eq. S8 to be evaluated. Overall, this allows the ID response to be written as in Eq. S9, where Φ_S is the Stern potential.(*37*)

$$\chi_{ID}^{(2)} = \int_0^{\infty} \left(\chi_D^{(2)} + \chi_D^{(3)} E_{DC}\right) e^{i\Delta k_z z} dz \tag{S7}$$

$$= \int_0^{z_S} \left(\chi_{D,CL}^{(2)} + +\chi_{D,CL}^{(3)} E_{DC}\right) e^{i\Delta k_z z} dz + \int_{z_S}^{\infty} \chi_{D,DL}^{(3)} E_{DC} e^{i\Delta k_z z} dz \tag{S8}$$

$$= \chi_{CL,eff}^{(2)} + \frac{1}{2}\Phi_S \chi_{DL}^{(3)} \cdot \left(1 + e^{2i \operatorname{atan}(\Delta k_z z_{DL})}\right) \tag{S9}$$

With this general description, the SFG and DFG responses for the two, oppositely charged surfactants (DHAB and DHP) can be written as in Eqs. S10-S13.

$$\chi_{DHAB,SFG}^{(2)} = \chi_{CL,DHAB}^{(2)} + \frac{1}{2}\Phi_{DHAB}\chi_{DL}^{(3)}\left(1 + e^{2i\phi_{SFG}}\right) + \chi_{IQ,DHAB}^{(2)} + \chi_{IQB,SFG}^{(2)} + \chi_{QB,SFG}^{(2)} \tag{S10}$$

$$\chi_{DHAB,DFG}^{(2)} = \chi_{CL,DHAB}^{(2)} + \frac{1}{2}\Phi_{DHAB}\chi_{DL}^{(3)}\left(1 + e^{2i\phi_{DFG}}\right) + \chi_{IQ,DHAB}^{(2)} + \chi_{IQB,DFG}^{(2)} + \chi_{QB,DFG}^{(2)} \tag{S11}$$

$$\chi_{DHP,SFG}^{(2)} = \chi_{CL,DHP}^{(2)} + \frac{1}{2}\Phi_{DHP}\chi_{DL}^{(3)}\left(1 + e^{2i\phi_{SFG}}\right) + \chi_{IQ,DHP}^{(2)} + \chi_{IQB,SFG}^{(2)} + \chi_{QB,SFG}^{(2)} \tag{S12}$$

$$\chi_{DHP,DFG}^{(2)} = \chi_{CL,DHP}^{(2)} + \frac{1}{2}\Phi_{DHP}\chi_{DL}^{(3)}\left(1 + e^{2i\phi_{DFG}}\right) + \chi_{IQ,DHP}^{(2)} + \chi_{IQB,DFG}^{(2)} + \chi_{QB,DFG}^{(2)} \tag{S13}$$

In these descriptions, the general quadrupolar contribution is broken down into its three contributors, based on the conventions of Morita(*4*), with IQ representing the anisotropic interfacial response, IQB being the bulk-like interfacial contribution, and QB being the fully isotropic bulk response. Furthermore, any parameter that is dependent on the surfactant is given a unique label. This includes the CL susceptibility, $\chi_{CL}^{(2)}$, which contains all of the chemistry-driven structural alterations due to the direct solvation of the surfactant, the Stern potential that is modulated by the surface charge density and specific dielectric structure in the CL, and the IQ quadrupolar response that is also dependent on

the molecular structure directly at the interface. Furthermore, as SFG and DFG have differing wavevector mismatches and potentially different quadrupolar amplitudes, they lead to unique contributions in the phase factor of the DL contribution, as well as both the QB and IQB contributions, as indicated by their labels.

Importantly, due to the sign-flip in charge of the two surfactants, $\chi_{CL}^{(2)}$ will have opposite signs for each, as will the two Stern potentials, Φ_{DHAB} and Φ_{DHP}. This means the overall ID response will flip sign between the two surfactants, but it may well have slightly different line-shapes owing to the surface-chemistry contributions in the CL contribution and likely different surface charged densities. By contrast, all the quadrupolar responses will have the same sign for both surfactants, but potentially a slightly different line-shape for the IQ response.

With these descriptions in hand, it becomes clear that combining all four responses can retrieve the bulk-like DL spectrum, as shown in Eq. S14, having corrected for the propagation phase associated with the Debye length.

$$\frac{1}{2}(\Phi_{\mathrm{DHAB}} - \Phi_{\mathrm{DHP}})\chi_{DL}^{(3)} = \frac{\chi_{DHAB,DFG}^{(2)} - \chi_{DHAB,SFG}^{(2)} - \chi_{DHP,DFG}^{(2)} + \chi_{DHP,SFG}^{(2)}}{e^{2i\phi_{DFG}} - e^{2i\phi_{SFG}}} \qquad (S14)$$

Subtraction of the Quadrupolar Contribution from Air-Water

As mentioned in the main text, both the experimental measurements presented in this work and recent theoretical predictions(*27*) indicate that the dominant contribution to the bending mode response is of quadrupolar origin and should present a bulk-like spectral line-shape. Specifically, the results from MD simulations, which do not consider QB contributions due to their inherent sensitivity to experimental settings, predict that the IQB contribution dominates. Using the formulations of Shen(*22*) and Morita(*4, 63, 64*), however, it has long been argued that the only experimentally separable quadrupolar contributions in SFG are the QB terms, e.g. using their coherence length scaling. This would imply that the amplitude difference between SFG and DFG can only arise from a dominant QB contribution. We recently showed(*27*), however, that the magnetic dipole contributions within the IQB term also give rise to a difference in amplitude between SFG and DFG. Therefore, while there are still slight differences between the experimental results and theoretical predictions(*27*), and with the latter omitting any QB contributions, both results indicate the dominant role of a bulk-like quadrupolar contribution to the bending spectrum.

As a result, the desired ID contribution to the pure air-water interface spectra can be isolated by subtracting the bulk-like DL line-shape obtained using Eq. S14 as it should have an identical line-shape to the bulk-like quadrupolar contribution. This, however, requires knowledge of the correct amount of the DL spectrum to subtract i.e., the absolute amplitude of the bulk-like quadrupolar contribution (which will generally be different to the DL contribution). As this contribution is also present for the charged interface responses, its amplitude can be estimated from these spectra. Specifically, the summation of the four spectra given by Eqs. S10-S13 should largely cancel the dipolar responses, only leaving residues due to their differences in specific chemistry-driven CL structure and Stern potentials. This means the total response should be dominated by the quadrupolar contributions that, as indicated in the main text, represent a significant part of the overall response. Therefore, by neglecting these slight dipolar residuals and the IQ contributions, the amplitude of the two bulk-like

quadrupolar responses (IQB and QB), which should be unchanged between the charged interface spectra and those at the pure air-water interface, can be estimated, and the appropriate amount of the DL line-shape subtracted. This approach was used to isolate the ID spectrum presented in the main text.

For further validation of this approach, one can also compare the simulated spectra to the residual experimental spectrum having subtracted different amplitudes of the DL line-shape. Such spectra are shown below in Figure S2, with the original SFG spectrum in red and the subtracted spectra in black (with the thicker black spectrum indicating the extracted ID contribution using the amplitude estimated from the charged interface spectra). With different subtracted amplitudes, it is clear that the overall line-shape highly differs, with these differences manifesting as changes in the zero-crossing and both the amplitudes and frequencies of the apparent 'dip' and 'peak' within the spectrum (or loss of either component). Therefore, a comparison between the different subtracted spectra and the calculated ID spectrum from the MD simulations gives three independent parameters to assess the quality of the match. On inspection of Figure S2 above as well as Figure 3 in the main text, the subtracted spectrum using the amplitude isolated from the charged interfaces yields exceptional overlap with all three of these parameters, far better than for any of the other presented subtractions in Figure S3. The quality of this match thus provides mutual validation of both the experimental and simulated spectra, suggesting that the desired experimental ID contribution is indeed isolated from this approach.

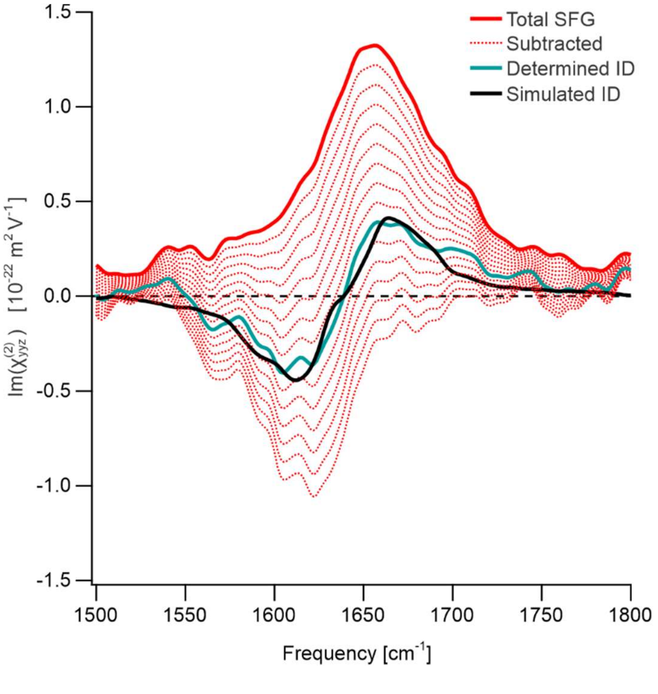

Figure S3: SFG spectra of the pure air-water interface having subtracted different amounts of the obtained DL spectrum. The original SFG spectrum is shown in solid red, the spectra with different subtractions in dotted red, the determined ID line-shape using the subtraction amplitude estimated from the charged interface spectra shown in solid turquoise, and the calculated ID spectrum from MD simulations in solid black.

Theoretical Prediction of the H-O-H Bending Response

If we take the molecular symmetry of each H_2O molecule as being C_{2v}, then there are 7 non-zero hyperpolarizability tensor elements: *aac*, *bbc*, *ccc*, *aca*=*caa*, and *bcb*=*cbb* (in the molecular frame coordinates (*a,b,c*) defined by the symmetry group, see Figure S4).

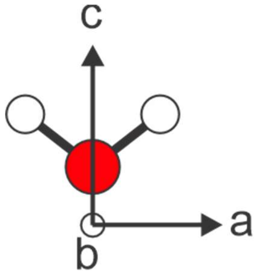

Figure S4: Molecular-frame coordinate of water.

For the H-O-H bending mode, which has the *A1* irreducible representation in C_{2v}, the relevant contributions are *aac*, *bbc*, and *ccc*, with the individual hyperpolarizability elements being given by Eq. S15, as the product of the partial derivatives of the Raman polarizability tensor, $\alpha^{(1)}$, and the IR transition dipole moment, μ, evaluated in the equilibrium geometry, where q represents the normal coordinate of the vibrational mode.(*65*)

$$\beta_{ijk} \propto \left(\frac{\partial \alpha_{ij}^{(1)}}{\partial q}\right)_0 \left(\frac{\partial \mu_k}{\partial q}\right)_0 \qquad (S15)$$

This shows that the three relevant components for the bending mode all involve the coupling of the IR along the dipole axis (*c*) and symmetric (diagonal) polarizabilities along each of the three molecular-frame coordinates.

The Euler transformation described by Eq. S16 can then be used to convert between the lab-frame and molecular-frame coordinates and isolate the *yyz* component of the second-order susceptibility.

$$\begin{pmatrix} x \\ y \\ z \end{pmatrix} = \begin{pmatrix} \cos\psi\cos\phi - \cos\theta\sin\phi\sin\psi & -\sin\psi\cos\phi - \cos\theta\sin\phi\cos\psi & \sin\theta\sin\phi \\ \cos\psi\sin\phi + \cos\theta\cos\phi\sin\psi & -\sin\psi\sin\phi + \cos\theta\cos\phi\cos\psi & -\sin\theta\cos\phi \\ \sin\theta\sin\psi & \sin\theta\cos\psi & \cos\theta \end{pmatrix} \begin{pmatrix} a \\ b \\ c \end{pmatrix} \qquad (S16)$$

If we then consider the in-plane Euler angle, ϕ, to be isotropically distributed, the *yyz* susceptibility component is given by Eq. S17 (given that $\langle \sin^2\phi \rangle = \langle \cos^2\phi \rangle = \frac{1}{2}$ and $\langle \sin\phi\cos\phi \rangle = 0$).

$$\chi_{yyz}^{(2)}(A_1) = \frac{1}{2} N[(\cos^2\psi\,\beta_{aac} + \sin^2\psi\,\beta_{bbc} + \beta_{ccc})\cos\theta + (\sin^2\psi\,\beta_{aac} + \cos^2\psi\,\beta_{bbc} - \beta_{ccc})\cos^3\theta] \qquad (S17)$$

To simplify Eq. S17, we then consider the relations between the three hyperpolarizability components. We first assume that the transition polarizability is dominated by the rotation of the OH-bond orbitals. Then, by denoting the OH bond polarizability tensor parallel to a OH-bond as $\alpha_{\parallel}$ and the perpendicular component as $\alpha_{\perp}$, and summing the polarizability contributions from the two OH-bond orbitals, the different components of the polarizability in the molecular frame can be expressed as in Eqs. S18-S20.

$$\alpha_{aa}(q) = 2\sin^2\left(\frac{\tau}{2}+q\right)\alpha_{\parallel} + 2\cos^2\left(\frac{\tau}{2}+q\right)\alpha_{\perp} \quad (S18)$$

$$\alpha_{bb}(q) = \alpha_{\perp} \quad (S19)$$

$$\alpha_{cc}(q) = 2\cos^2\left(\frac{\tau}{2}+q\right)\alpha_{\parallel} + 2\sin^2\left(\frac{\tau}{2}+q\right)\alpha_{\perp} \quad (S20)$$

In these expressions, the vibrational coordinate, q, for the bending mode is considered as simply an angular change to the dihedral angle of the two O-H bonds, $\tau \approx 104°$.

For the bending mode, we know that $\left(\frac{\partial\mu_i}{\partial q}\right)_0 = \delta_{ic}\Delta\mu$, where $\Delta\mu$ is the transition dipole moment. This leads to the expressions for the three components of the molecular hyperpolarizability given in Eqs. S21-S23.

$$\beta_{aac} = \left(\frac{\partial\alpha_{aa}^{(1)}}{\partial q}\right)_0 \left(\frac{\partial\mu_c}{\partial q}\right)_0 = 2\sin\tau\,(\alpha_{\parallel} - \alpha_{\perp})\,\Delta\mu \quad (S21)$$

$$\beta_{bbc} = \left(\frac{\partial\alpha_{bb}^{(1)}}{\partial q}\right)_0 \left(\frac{\partial\mu_c}{\partial q}\right)_0 = 0\cdot\Delta\mu \quad (S22)$$

$$\beta_{ccc} = \left(\frac{\partial\alpha_{cc}^{(1)}}{\partial q}\right)_0 \left(\frac{\partial\mu_c}{\partial q}\right)_0 = -2\sin\tau\,(\alpha_{\parallel} - \alpha_{\perp})\,\Delta\mu \quad (S23)$$

These expressions, which are purely derived from geometric arguments, can be further validated by comparison to calculated values of the polarisability derivatives. For the bending mode, using the rotating bond orbital approximation, calculations of the changes in the polarizability tensor in response to an angular change of $\Delta\tau = -0.0158^{\circ}$ yielded the non-zero elements shown in Eq. S24.

$$\Delta\alpha_{ij} = \begin{pmatrix} -2.24 & 0 & 0 \\ 0 & -0.19 & 0 \\ 0 & 0 & 2.56 \end{pmatrix} \times 10^{-3}\,\text{Å}^3 \quad (S24)$$

These values, as well as those from more sophisticated calculations of the polarizability derivate in the literature(*66*), clearly agree well with the above approximation for the relations between the relevant hyperpolarizability components.

With these approximations, Eq. S17 can be rewritten as in Eq. S25.

$$\chi^{(2)}_{yyz}(A_1) = \frac{1}{2}N\beta_{ccc}\cos\theta\,[(1-\cos^2\psi) \\ -(1+\sin^2\psi)\cos^2\theta] \\ = \frac{1}{2}N\beta_{ccc}\cos\theta\,[\sin^2\theta\,(1+\sin^2\psi)-1] \qquad (S25)$$

Figure 5b in the main text shows the calculation of the *yyz* component of the second-order susceptibility using the expression in Eq. S25, being given relative to the *ccc* component of the molecular hyperpolarizability and as a function of the molecular tilt and twist angles.

REFERENCES

1. O. Björneholm, M. H. Hansen, A. Hodgson, L.-M. Liu, D. T. Limmer, A. Michaelides, P. Pedevilla, J. Rossmeisl, H. Shen, G. Tocci, E. Tyrode, M.-M. Walz, J. Werner, H. Bluhm, Water at interfaces. *Chem. Rev.* **116**, 7698–7726 (2016).

2. M. Falk, The frequency of the HOH bending fundamental in solids and liquids. *Spectrochim. Acta A* **40**, 43–48 (1984).

3. T. Seki, K.-Y. Chiang, C.-C. Yu, X. Yu, M. Okuno, J. Hunger, Y. Nagata, M. Bonn, The bending mode of water: A powerful probe for hydrogen bond structure of aqueous systems. *J. Phys. Chem. Lett.* **11**, 8459–8469 (2020).

4. A. Morita, *Theory of Sum Frequency Generation Spectroscopy* (Springer, 2018).

5. Y. R. Shen, *Fundamentals of Sum-Frequency Spectroscopy*, Cambridge Molecular Science (Cambridge Univ. Press, 2016).

6. R. W. Boyd, *Nonlinear Optics* (Elsevier Inc., ed. 4, 2020).

7. A. G. Lambert, P. B. Davies, D. J. Neivandt, Implementing the theory of sum frequency generation vibrational spectroscopy: A tutorial review. *Appl. Spectrosc. Rev.* **40**, 103–145 (2005).

8. Y. R. Shen, Phase-sensitive sum-frequency spectroscopy. *Annu. Rev. Phys. Chem.* **64**, 129–150 (2013).

9. T. Ishiyama, A. Morita, Computational analysis of vibrational sum frequency generation spectroscopy. *Annu. Rev. Phys. Chem.* **68**, 355–377 (2017).

10. M. Sulpizi, M. Salanne, M. Sprik, M.-P. Gaigeot, Vibrational sum frequency generation spectroscopy of the water liquid–vapor interface from density functional theory-based molecular dynamics simulations. *J. Phys. Chem. Lett.* **4**, 83–87 (2013).

11. A. Perry, H. Ahlborn, B. Space, P. B. Moore, A combined time correlation function and instantaneous normal mode study of the sum frequency generation spectroscopy of the water/vapor interface. *J. Chem. Phys.* **118**, 8411–8419 (2003).

12. A. Morita, J. T. Hynes, A theoretical analysis of the sum frequency generation spectrum of the water surface. II. Time-dependent approach. *J. Phys. Chem. B* **106**, 673–685 (2002).

13. A. Perry, C. Neipert, B. Space, P. B. Moore, Theoretical modeling of interface specific vibrational spectroscopy: Methods and applications to aqueous interfaces. *Chem. Rev.* **106**, 1234–1258 (2006).

14. T. Ishiyama, T. Imamura, A. Morita, Theoretical studies of structures and vibrational sum frequency generation spectra at aqueous interfaces. *Chem. Rev.* **114**, 8447–8470 (2014).

15. A. Morita, Toward computation of bulk quadrupolar signals in vibrational sum frequency generation spectroscopy. *Chem. Phys. Lett.* **398**, 361–366 (2004).

16. B. M. Auer, J. L. Skinner, Vibrational sum-frequency spectroscopy of the liquid/vapor interface for dilute HOD in D_2O. *J. Chem. Phys.* **129**, 214705 (2008).

17. D. S. Walker, D. K. Hore, G. L. Richmond, Understanding the population, coordination, and orientation of water species contributing to the nonlinear optical spectroscopy of the vapor–water interface through molecular dynamics simulations. *J. Phys. Chem. B* **110**, 20451–20459 (2006).

18. F. Tang, T. Ohto, S. Sun, J. R. Rouxel, S. Imoto, E. H. G. Backus, S. Mukamel, M. Bonn, Y. Nagata, Molecular structure and modeling of water–air and ice–air interfaces monitored by sum-frequency generation. *Chem. Rev.* **120**, 3633–3667 (2020).

19. J. Schaefer, E. H. G. Backus, Y. Nagata, M. Bonn, Both inter- and intramolecular coupling of O–H groups determine the vibrational response of the water/air interface. *J. Phys. Chem. Lett.* **7**, 4591–4595 (2016).

20. D. R. Moberg, S. C. Straight, F. Paesani, Temperature dependence of the air/water interface revealed by polarization sensitive sum-frequency generation spectroscopy. *J. Phys. Chem. B* **122**, 4356–4365 (2018).

21. H. J. Bakker, J. L. Skinner, Vibrational spectroscopy as a probe of structure and dynamics in liquid water. *Chem. Rev.* **110**, 1498–1517 (2010).

22. Y. R. Shen, Revisiting the basic theory of sum-frequency generation. *J. Chem. Phys.* **153**, 180901 (2020).

23. H.-F. Wang, Sum frequency generation vibrational spectroscopy (SFG-VS) for complex molecular surfaces and interfaces: Spectral lineshape measurement and analysis plus some controversial issues. *Prog. Surf. Sci.* **91**, 155–182 (2016).

24. A. P. Fellows, V. Balos, B. John, Á. Díaz Duque, M. Wolf, M. Thämer, Obtaining extended insight into molecular systems by probing multiple pathways in second-order nonlinear spectroscopy. *J. Chem. Phys.* **159**, 164201 (2023).

25. V. Balos, T. Garling, A. D. Duque, B. John, M. Wolf, M. Thämer, Phase-sensitive vibrational sum and difference frequency-generation spectroscopy enabling nanometer-depth profiling at interfaces. *J. Phys. Chem. C* **126**, 10818–10832 (2022).

26. A. P. Fellows, Á. D. Duque, V. Balos, L. Lehmann, R. R. Netz, M. Wolf, M. Thämer, How thick is the air–water interface?—A direct experimental measurement of the decay length of the interfacial structural anisotropy. *Langmuir* **40**, 18760–18772 (2024).

27. L. Lehmann, M. R. Becker, L. Tepper, A. P. Fellows, Á. D. Duque, M. Thämer, R. R. Netz, Beyond the electric dipole approximation: Electric and magnetic multipole contributions reveal biaxial water structure from SFG spectra at the air-water interface. arXiv:2505.19856 [cond-mat.stat-mech] (2025).

28. Y. Nagata, C.-S. Hsieh, T. Hasegawa, J. Voll, E. H. G. Backus, M. Bonn, Water bending mode at the Water–Vapor interface probed by sum-frequency generation spectroscopy: A combined

molecular dynamics simulation and experimental study. *J. Phys. Chem. Lett.* **4**, 1872–1877 (2013).

29. Y. Ni, J. L. Skinner, IR and SFG vibrational spectroscopy of the water bend in the bulk liquid and at the liquid-vapor interface, respectively. *J. Chem. Phys.* **143**, 014502 (2015).

30. M. Vinaykin, A. V. Benderskii, Vibrational sum-frequency spectrum of the water bend at the air/water interface. *J. Phys. Chem. Lett.* **3**, 3348–3352 (2012).

31. M. Ahmed, S. Nihonyanagi, A. Kundu, S. Yamaguchi, T. Tahara, Resolving the controversy over dipole versus quadrupole mechanism of bend vibration of water in vibrational sum frequency generation spectra. *J. Phys. Chem. Lett.* **11**, 9123–9130 (2020).

32. A. Kundu, S. Tanaka, T. Ishiyama, M. Ahmed, K. Inoue, S. Nihonyanagi, H. Sawai, S. Yamaguchi, A. Morita, T. Tahara, Bend vibration of surface water investigated by heterodyne-detected sum frequency generation and theoretical study: Dominant role of quadrupole. *J. Phys. Chem. Lett.* **7**, 2597–2601 (2016).

33. C. J. Moll, J. Versluis, H. J. Bakker, Direct evidence for a surface and bulk specific response in the sum-frequency generation spectrum of the water bend vibration. *Phys. Rev. Lett.* **127**, 116001 (2021).

34. T. Seki, S. Sun, K. Zhong, C. C. Yu, K. MacHel, L. B. Dreier, E. H. G. Backus, M. Bonn, Y. Nagata, Unveiling heterogeneity of interfacial water through the water bending mode. *J. Phys. Chem. Lett.* **10**, 6936–6941 (2019).

35. T. Seki, C.-C. Yu, X. Yu, T. Ohto, S. Sun, K. Meister, E. H. G. Backus, M. Bonn, Y. Nagata, Decoding the molecular water structure at complex interfaces through surface-specific spectroscopy of the water bending mode. *Phys. Chem. Chem. Phys.* **22**, 10934–10940 (2020).

36. T. Seki, C. C. Yu, K. Y. Chiang, J. Tan, S. Sun, S. Ye, M. Bonn, Y. Nagata, Disentangling sum-frequency generation spectra of the water bending mode at charged aqueous interfaces. *J. Phys. Chem. B* **125**, 7060–7067 (2021).

37. A. P. Fellows, Á. D. Duque, V. Balos, L. Lehmann, R. R. Netz, M. Wolf, M. Thämer, Sum-frequency generation spectroscopy of aqueous interfaces: The role of depth and its impact on spectral interpretation. *J. Phys. Chem. C* **128**, 20733–20750 (2024).

38. E. C. Y. Yan, Y. Liu, K. B. Eisenthal, New method for determination of surface potential of microscopic particles by second harmonic generation. *J. Phys. Chem. B* **102**, 6331–6336 (1998).

39. F. M. Geiger, Second harmonic generation, sum frequency generation, and $\chi^{(3)}$: Dissecting environmental interfaces with a nonlinear optical Swiss Army knife. *Annu. Rev. Phys. Chem.* **60**, 61–83 (2009).

40. K. C. Jena, P. A. Covert, D. K. Hore, The effect of salt on the water structure at a charged solid surface: Differentiating second- and third-order nonlinear contributions. *J. Phys. Chem. Lett.* **2**, 1056–1061 (2011).

41. G. Gonella, C. Lütgebaucks, A. G. F. de Beer, S. Roke, Second harmonic and sum-frequency generation from aqueous interfaces is modulated by interference. *J. Phys. Chem. C* **120**, 9165–9173 (2016).

42..D iaz-Duque, V. Balos, M. Wolf, A. P. Fellows, M. Thämer, Anisotropic water structure at charged interfaces studied by depth resolved vibrational SFG/DFG spectroscopy. arXiv:2508.06912 [physics.chem-ph] (2025).

43. Y.-C. Wen, S. Zha, X. Liu, S. Yang, P. Guo, G. Shi, H. Fang, Y. R. Shen, C. Tian, Unveiling microscopic structures of charged water interfaces by surface-specific vibrational spectroscopy. *Phys. Rev. Lett.* **116**, 016101 (2016).

44. Y. Hsiao, T.-H. Chou, A. Patra, Y.-C. Wen, Momentum-dependent sum-frequency vibrational spectroscopy of bonded interface layer at charged water interfaces. *Sci. Adv.* **9**, eadg2823 (2024).

45.. Díaz Duque, S. Kaur, M. Wolf, A. P. Fellows, M. G. Thämer, The influence of electrolyte concentration on the depth-dependent structural anisotropy of water at charged interfaces. *Faraday Discuss.* **2026**, 10.1039/D5FD00155B (2026).

46. R. Oder, D. A. I. Goring, An overtone of the infrared librational absorption in liquid water. *Spectrochim. Acta A* **27**, 2285–2288 (1971).

47. C. Dutta, A. V. Benderskii, On the assignment of the vibrational spectrum of the water bend at the air/water interface. *J. Phys. Chem. Lett.* **8**, 801–804 (2017).

48. P. A. Pieniazek, C. J. Tainter, J. L. Skinner, Interpretation of the water surface vibrational sum-frequency spectrum. *J. Chem. Phys.* **135**, 044701 (2011).

49. P. E. Ohno, H. Wang, F. M. Geiger, Second-order spectral lineshapes from charged interfaces. *Nat. Commun.* **8**, 1032 (2017).

50. W. Sung, K. Inoue, S. Nihonyanagi, T. Tahara, Unified picture of vibrational relaxation of OH stretch at the air/water interface. *Nat. Commun.* **15**, 1258 (2024).

51. S. Pezzotti, D. R. Galimberti, M.-P. Gaigeot, 2D H-bond network as the topmost skin to the air–water interface. *J. Phys. Chem. Lett.* **8**, 3133–3141 (2017).

52. F. Sedlmeier, J. Janecek, C. Sendner, L. Bocquet, R. R. Netz, D. Horinek, Water at polar and nonpolar solid walls (Review). *Biointerphases* **3**, FC23–FC39 (2008).

53. J. Kessler, H. Elgabarty, T. Spura, K. Karhan, P. Partovi-Azar, A. A. Hassanali, T. D. Kühne, Structure and dynamics of the instantaneous water/vapor interface revisited by path-integral and ab initio molecular dynamics simulations. *J. Phys. Chem. B* **119**, 10079–10086 (2015).

54. S. Shin, A. P. Willard, Three-body hydrogen bond defects contribute significantly to the dielectric properties of the liquid water–vapor interface. *J. Phys. Chem. Lett.* **9**, 1649–1654 (2018).

55. Q. Du, R. Superfine, E. Freysz, Y. R. Shen, Vibrational spectroscopy of water at the vapor/water interface. *Phys. Rev. Lett.* **70**, 2313–2316 (1993).

56. P. Jedlovszky, Á. Vincze, G. Horvai, New insight into the orientational order of water molecules at the water/1,2-dichloroethane interface: A Monte Carlo simulation study. *J. Chem. Phys.* **117**, 2271–2280 (2002).

57. S. Pezzotti, A. Serva, M.-P. Gaigeot, 2D-HB-Network at the air-water interface: A structural and dynamical characterization by means of ab initio and classical molecular dynamics simulations. *J. Chem. Phys.* **148**, 174701 (2018).

58. F. Sedlmeier, D. Horinek, R. R. Netz, Nanoroughness, intrinsic density profile, and rigidity of the air-water interface. *Phys. Rev. Lett.* **103**, 136102 (2009).

59. F. N. Brünig, O. Geburtig, A. von Canal, J. Kappler, R. R. Netz, Time-dependent friction effects on vibrational infrared frequencies and line shapes of liquid water. *J. Phys. Chem. B* **126**, 1579–1589 (2022).

60. A. P. Fellows, M. T. L. Casford, P. B. Davies, Investigating Bénard–Marangoni migration at the air–water interface in the time domain using sum frequency generation (SFG) spectroscopy of palmitic acid monolayers. *J. Chem. Phys.* **156**, 164701 (2022).

61. M. Thämer, R. K. Campen, M. Wolf, Detecting weak signals from interfaces by high accuracy phase-resolved SFG spectroscopy. *Phys. Chem. Chem. Phys.* **20**, 25875–25882 (2018).

62. M. Thämer, T. Garling, R. K. Campen, M. Wolf, Quantitative determination of the nonlinear bulk and surface response from alpha-quartz using phase sensitive SFG spectroscopy. *J. Chem. Phys.* **151**, 64707 (2019).

63. T. Hirano, A. Morita, Boundary effects and quadrupole contribution in sum frequency generation spectroscopy. *J. Chem. Phys.* **156**, 154109 (2022).

64. T. Hirano, A. Morita, Local field effects of quadrupole contributions on sum frequency generation spectroscopy. *J. Chem. Phys*. **161**, (2024).

65. H. F. Wang, W. Gan, R. Lu, Y. Rao, B. H. Wu, Quantitative spectral and orientational analysis in surface sum frequency generation vibrational spectroscopy (SFG-VS). *Int. Rev. Phys. Chem.* **24**, 191–256 (2005).

66. K. U. Lao, J. Jia, R. Maitra, R. A. DiStasio, On the geometric dependence of the molecular dipole polarizability in water: A benchmark study of higher-order electron correlation, basis

set incompleteness error, core electron effects, and zero-point vibrational contributions. *J. Chem. Phys.* **149**, (2018).
